# Prurigo

**Published:** 1857-02

**Authors:** 


*Prurigo.*

During the past few months, three cases of Prurigo formicans have been admitted into the Mercy Hospital. The disease affected chiefly the whole posterior part of the trunk, and the arms. It had existed several weeks before admission, and was attended, as usual, with itching so intolerable as to deprive the patients of sleep during most of the night.

These cases were treated with Fowler's Solution of Arsenic, from six to ten drops three times a-day, and an occasional laxative internally. At the same time the following ointment was freely applied to the surface each morning and evening:—

℞—Iodide of Potassa, . . ℥ss.

Simple Cerate, . . ℥iv.

Mix very thoroughly.

The patients were discharged apparently quite well, at times varying from one to two weeks. The relief seemed to be derived chiefly from the external application of the ointment, which certainly acted more promptly and beneficially than any other local application that had been used in other cases.

